# Supplementary material for: Health related quality of life of people receiving highly active antiretroviral therapy in Southwest Ethiopia
Source: PLoS One. 2020 Aug 20;15(8):e0237013. doi: 10.1371/journal.pone.0237013 (PMC7446831; doi:10.1371/journal.pone.0237013)
Supplement: S1 Data — (DOCX) [file pone.0237013.s001.docx]

# Data collection tool

**Questionnaire**

**Part I: Sociodemographic Characteristics**

1. Sex
2. Male
3. Female
4. Age (years)________
5. Educational level
6. Cannot read and write
7. Primary (grades 1-8)
8. Secondary (grades 9-12)
9. Tertiary (TVET/College/University)
10. Marital status
11. Single
12. Married
13. Divorced
14. Widowed
15. Type of occupation/employment
16. Farmer
17. Gov’t Employee
18. Trade/private business
19. Daily laborer
20. Housewife
21. Retired
22. Unemployed
23. Other______
24. Residence
25. Rural
26. Urban
27. Do you have any children?
28. Yes
29. No

**Part II: Self-perceived Health Status and Health related information**

1. How do you rate your current status of health?
2. Very Poor
3. Poor
4. Neither Poor nor Good
5. Good
6. Very Good
7. Do you consider yourself currently ill?
8. Yes
9. No
10. How do you believe you were infected with HIV?
11. Sexual intercourse
12. Injecting drugs
13. Blood products
14. Unknown
15. Other, specify
16. How long it is now since tested positive for HIV? (months)__________
17. Have you disclosed your HIV status to relatives?
18. Yes
19. No
20. How many family members are affected with HIV (Seropositive status)? ____
21. How do you rate your living environment?
22. Very bad
23. Bad
24. Neither bad nor good
25. Good
26. Very good
27. Is there any family support for you?
28. Yes
29. No
30. How do you rate your social relationship?
31. Very bad
32. Bad
33. Neither bad nor good
34. Good
35. Very good
36. What is your perceived quality of life before starting to take ART medications?
37. Very bad
38. Bad
39. Neither bad nor good
40. Good
41. Very good
42. Have you ever used any substance?
43. Yes
44. No
45. Are you currently using any substance?
46. Yes
47. No
48. If your response for question number 19 is yes, what type of substance you are currently using? (can circle more than once)
49. Alcohol
50. Khat
51. Cigarette
52. Other_________
53. How many times you take your ART medications per day ______?
54. Do you have regular follow up for your HIV?
55. Yes
56. No
57. How do you rate your relationship with your health care provider? (Trust and satisfaction with information)
58. Very bad
59. Bad
60. Neither bad nor good
61. Good
62. Very good

**Part III: ART Medication Adherence (Morisky Medication Adherence Scale, MMAS-8)**

**(Patients are expected to remember for the last 2 weeks)**

| S.No | Questions | Response | |
| --- | --- | --- | --- |
|  |  | Yes (1) | No (0) |
|  | Do you sometimes forget to take your ART pills? |  |  |
|  | People sometimes miss taking their medications for reasons other than forgetting. Thinking over the past two weeks, were there any days when you did not take your ART medications? |  |  |
|  | Have you ever cut back or stopped taking your ART medication without telling your doctor because you felt worse when you took it? |  |  |
|  | When you travel or leave home, do you sometimes forget to bring along your ART medication? |  |  |
|  | Did you take your ART medication yesterday? |  |  |
|  | When you feel like your glucose level is under control, do you sometimes stop taking your ART medication? |  |  |
|  | Taking medication every day is a real inconvenience for some people. Do you ever feel hassled about sticking to your ART treatment plan? |  |  |
|  | How often do you have difficulty remembering to take all your ART medications?   - 1. Never/rarely (0)   2. Once in a while (1)   3. Sometimes (2)   4. Usually (3)   5. All the time (4)   **Score (out of 8): ___________** | | |

Positive responses=0; Negative responses=1

**Part IV: Assessment of Quality of Life: WHOQOL-BREF Tool**

| S.No | Question | Very Poor | | Poor | | | | | Neither Poor nor Good | | | | | | Good | | | Very Good |
| --- | --- | --- | --- | --- | --- | --- | --- | --- | --- | --- | --- | --- | --- | --- | --- | --- | --- | --- |
|  | How do you rate your QOL? |  | |  | | | | |  | | | | | |  | | |  |
| S.No | Question | Very dissatisfied | | Dissatisfied | | | | | Neither satisfied nor dissatisfied | | | | | | Satisfied | | | Very satisfied |
|  | How satisfied are you with your health? | 1 | | 2 | | | | | 3 | | | | | | 4 | | | 5 |
| The following questions ask about how **much you** have experienced certain things in the last 2 weeks | | | | | | | | | | | | | | | | | | |
| S.No | Question | | | | | Not at all | | A little | | | | A moderate amount | | Very much | | An extreme amount | | |
|  | To what extent do you feel that physical pain prevents you from doing what you need to do? | | | | | 1 | | 2 | | | | 3 | | 4 | | 5 | | |
|  | How much are you bothered by any physical problems related to your HIV infection? | | | | | 1 | | 2 | | | | 3 | | 4 | | 5 | | |
|  | How much do you need any medical treatment to function in your daily life? | | | | | 1 | | 2 | | | | 3 | | 4 | | 5 | | |
|  | How much do you enjoy life? | | | | | 1 | | 2 | | | | 3 | | 4 | | 5 | | |
|  | To what extent do you feel your life to be meaningful? | | | | | 1 | | 2 | | | | 3 | | 4 | | 5 | | |
|  | To what extent are you bothered by people blaming you for your HIV status? | | | | | 1 | | 2 | | | | 3 | | 4 | | 5 | | |
|  | How much do you fear the future? | | | | | 1 | | 2 | | | | 3 | | 4 | | 5 | | |
|  | How much do you worry about death? | | | | | 1 | | 2 | | | | 3 | | 4 | | 5 | | |
|  | How well are you able to concentrate? | | | | | 1 | | 2 | | | | 3 | | 4 | | 5 | | |
|  | How safe do you feel in your daily life? | | | | | 1 | | 2 | | | | 3 | | 4 | | 5 | | |
|  | How healthy is your physical environment? | | | | | 1 | | 2 | | | | 3 | | 4 | | 5 | | |
| The following questions ask about **how completely** you experience or were able to do certain things in the last two weeks | | | | | | | | | | | | | | | | | | |
| S.No | Question | | | Not at all | | | A little | | | | Moderately | | Mostly | | | Completely | | |
|  | Do you have enough energy for everyday life? | | | 1 | | | 2 | | | | 3 | | 4 | | | 5 | | |
|  | Are you able to accept your bodily appearance? | | | 1 | | | 2 | | | | 3 | | 4 | | | 5 | | |
|  | Have you enough money to meet your days? | | | 1 | | | 2 | | | | 3 | | 4 | | | 5 | | |
|  | To what extent do you feel accepted by the people you know? | | | 1 | | | 2 | | | | 3 | | 4 | | | 5 | | |
|  | How available to you is the information that you need in your day-to-day life? | | | 1 | | | 2 | | | | 3 | | 4 | | | 5 | | |
|  | To what extent do you have the opportunity for leisure activities? | | | 1 | | | 2 | | | | 3 | | 4 | | | 5 | | |
| S.No | Question | | | Very poor | | | Poor | | | | Neither Poor nor Good | | Good | | | Very Good | | |
|  | How well are you able to get around? | | |  | | |  | | | |  | |  | | |  | | |
| The following questions ask you how good or satisfied you have felt about various aspects of your life over the last two weeks | | | | | | | | | | | | | | | | | | |
| S.No | Question | | Very satisfied | | Dissatisfied | | | | | Neither satisfied nor dissatisfied | | | | Satisfied | | | Very satisfied | |
|  | How satisfied are you with your sleep? | | 1 | | 2 | | | | | 3 | | | | 4 | | | 5 | |
|  | How satisfied are you with your ability to perform your daily living activities? | | 1 | | 2 | | | | | 3 | | | | 4 | | | 5 | |
|  | How satisfied are you with your capacity for work? | | 1 | | 2 | | | | | 3 | | | | 4 | | | 5 | |
|  | How satisfied are you with yourself? | | 1 | | 2 | | | | | 3 | | | | 4 | | | 5 | |
|  | How satisfied are you with your personal relationships? | | 1 | | 2 | | | | | 3 | | | | 4 | | | 5 | |
|  | How satisfied are you with your sex life? | | 1 | | 2 | | | | | 3 | | | | 4 | | | 5 | |
|  | How satisfied are you with the support you get from your friends? | | 1 | | 2 | | | | | 3 | | | | 4 | | | 5 | |
|  | How satisfied are you with the conditions of your living place? | | 1 | | 2 | | | | | 3 | | | | 4 | | | 5 | |
|  | How satisfied are you with your access to health services? | | 1 | | 2 | | | | | 3 | | | | 4 | | | 5 | |
|  | How satisfied are you with your transport? | | 1 | | 2 | | | | | 3 | | | | 4 | | | 5 | |
| The following question refers to **how often** you have felt or experienced certain things in the last two weeks. | | | | | | | | | | | | | | | | | | |
| S.No | Question | | Never | | Seldom | | | | | Quite often | | | | Very often | | | Always | |
|  | How often do you have negative feelings such as blue mood, despair, anxiety, depression? | | 1 | | 2 | | | | | 3 | | | | 4 | | | 5 | |

**QoL among PLWHA on HAART at MTUTH CHART EXTRACTION FORMAT**

**Clinical Information**

1. Age at ART initiation (Years) ______________
2. Weight (Kg):
3. Baseline________
4. Current____________
5. Current Height (Cm)_________ Current BMI (Kg/m^2^) _________
6. WHO Clinical Stage (Stages I/II/III/IV):
7. Baseline___________
8. Current ______________
9. CD_4_ Count (cells/mm^3^):
10. Baseline ____________
11. Current____________
12. Viral Load(copies/ml):
13. Baseline___________
14. Current_______________
15. Cotrimoxazole Preventive Therapy (CPT)
16. Yes
17. No
18. Isoniazid Preventive Therapy (IPT)
19. Yes
20. No
21. Type of initial HAART regimen __________
22. Total duration on HAART (Months)___________
23. Was there an initial Regimen change?
24. Yes
25. No
26. If yes for question number 73, what was the reason for regimen change? _____
27. Was there any Comorbidity?
28. Yes
29. No
30. If yes for question number 75, what was the type of comorbidity? _________
31. Was there any Opportunistic Infections?
32. Yes
33. No
34. If yes for question number 77, what was the type of opportunistic infection? _______
35. Were there any adverse drug reaction encountered?
36. Yes
37. No

**Amharic version**

**ቃለ-መጠይቅ**

**ምዕራፍ አንድ**

1. ጾታ
2. ወንድ
3. ሴት
4. ዕድሜ (በዓመት)___________
5. የትምህርት ደረጃ
6. መፃፍና ማንበብ የማይችል/የማትችል/
7. አንደኛ ደረጃ(1-8ኛ)
8. ሁለተኛ ደረጃ(9-12ኛ)
9. ኮሌጅ የተማረ/የተማረች/
10. የጋብቻ ሁኔታ
11. ያላገባ/ች/
12. ያገባ/ች/
13. የተፋታ/የተፋታች/
14. ሚስቱ የሞተችበት/ባሏ የሞተባት/
15. የስራ ሁኔታ
16. አርሶ አደር
17. የመንግስት ሠራተኛ
18. የንግድ ስራ
19. የቀን ሰራተኛ
20. የቤት እመቤት
21. ጡረተኛ
22. ስራ የሌለው/የሌላት/
23. ሌላ ________
24. የመኖሪያ ቦታ
25. ገጠር
26. ከተማ
27. ልጆች አሉዎት?
28. አዎ
29. የሉኝም

**ምዕራፍ ሁለት**

1. አሁን ያሉበትን የጤና ሁኔታ እንዴት ይገልጹታል?
2. በጣም መጥፎ
3. መጥፎ
4. ጥሩም መጥፎም ያልሆነ
5. ጥሩ
6. በጣም ጥሩ
7. ራስዎን በአሁኑ ሰዓት ታማሚ አድርገው ያስባሉ?
8. አዎ
9. አላስብም
10. HIV እንዴት ባለ ሁኔታ እንደያዘዎት ያምናሉ?
11. በግብረ ስጋ ግንኙነት
12. በደም ንክኪ
13. አላውቅም
14. በሌላ____________
15. ለHIV ፖዚቲቭ ከሆኑ ምን ያህል ጊዜ ሆነዎት? (በወራት) ____________
16. HIV እንዳለብዎ ቤተሰቦችዎ ያውቃሉ?
17. አዎ
18. አያውቁም
19. ከእርስዎ ውጭ HIV ያለበት የቤተሰብ አባል ስንት ነው? _____________
20. የሚኖሩበትን የኑሮ ሁኔታ እንዴት ይገልጹታል?
21. በጣም መጥፎ
22. መጥፎ
23. ጥሩም መጥፎም ያልሆነ
24. ጥሩ
25. በጣም ጥሩ
26. የቤተሰብ ድጋፍ ያገኛሉ?
27. አዎ
28. አላገኝም
29. ከሰዎች ጋር ያለዎትን ማህበራዊ ግንኙነት እንዴት ይገልፁታል?
30. በጣም መጥፎ
31. መጥፎ
32. ጥሩም መጥፎም ያልሆነ
33. ጥሩ
34. በጣም ጥሩ
35. ለህመምዎ መድሃኒት መውሰድ ከመጀመርዎ በፊት ያለውን የኑሮዎን ሁኔታ እንዴት ይገልፁታል?
36. በጣም መጥፎ
37. መጥፎ
38. ጥሩም መጥፎም ያልሆነ
39. ጥሩ
40. በጣም ጥሩ
41. ሱስ የሚያስይዙ ነገሮችን ተጠቅመው ያውቃሉ?
42. አዎ
43. ተጠቅሜ አላውቅም
44. ለጥያቄ ቁጥር 18 ምላሽዎ አዎ የሚል ከሆነ በአሁኑ ሰዓትስ ሱስ የሚያስይዙ ነገሮችን ይጠቀማሉ?
45. አዎ
46. አልጠቀምም
47. ለጥያቄ ቁጥር 19 ምላሽዎ አዎ የሚል ከሆነ ምን አይነት ሱስ የሚያስይዝ ነገር/ዕፅ/ ይጠቀማሉ?
48. አልኮል
49. ጫት
50. ሲጋራ
51. ሌላ ___________
52. የHIV መድሃኒትዎን በቀን ስንት ጊዜ ይወስዳሉ? _______
53. ለህመምዎ መደበኛ የሆነ የህክምና ክትትል ያደርጋሉ?
54. አዎ
55. አላደርግም
56. ከጤና ባለሙያዎች ጋር ያለዎትን ግንኙነት እንዴት ይገልፁታል?
57. በጣም መጥፎ
58. መጥፎ
59. ጥሩም መጥፎም ያልሆነ
60. ጥሩ
61. በጣም ጥሩ

**ምዕራፍ ሶስት፡ MMAPS-8 (ባለፉት 2 ሳምንታት ውስጥ)**

| **ተ.ቁ.** | **ጥያቄ** | **ምላሽ** | |
| --- | --- | --- | --- |
|  |  | አዎ (1) | አይ (0) |
|  | አንዳንድ ጊዜ መድሃኒትዎን መውሰድ ረስተው ያውቃሉ? |  |  |
|  | ሰዎች አንዳንድ ጊዜ መድሃኒታቸውን ከመርሳት ውጭ ባለ ምክንያት ላይወስዱ ይችላሉ፡፡ ባለፉት ሁለት ሳምንታት ውስጥ መድሃኒትዎን ሳይወስዱ የቀሩበት ቀን አለ? |  |  |
|  | መድሃኒትዎን እየወሰዱ ህመምዎ ስለተባባሰብዎ ሀኪምዎን ሳያማክሩ መድሃኒትዎን አቋርጠው ያውቃሉ? |  |  |
|  | ከቤት ርቀው ጉዞ ሲወጡ አንዳንድ ጊዜ መድሃኒትዎን ከእርስዎ ጋር መውሰድ ረስተው ያውቃሉ? |  |  |
|  | መድሃኒትዎን ትናንት ወስደዋል? |  |  |
|  | አንዳንድ ጊዜ ህመምዎን የተቆጣጠሩ ሲመስልዎት መድሃኒትዎን ሳይወስዱ የቀሩበት ጊዜ አለ? |  |  |
|  | አንዳንድ ሰዎች መድሃኒታቸውን በየቀኑ መውሰድ ላይመቻቸው ይችላል፡፡ ህክምናዎን በደንብ ለመከታተል ምቾት አጥተው ያውቃሉ? |  |  |
|  | ምን ያህል ጊዜ ሁሉንም የHIV ህመም መድሃኒትዎን ለመውሰድ በመርሳት ተቸግረው ያውቃሉ?   - 1. ተቸግሬ አላውቅም (0)   2. አንድ ጊዜ ብቻ (1)   3. አንዳንድ ጊዜ (2)   4. ብዙ ጊዜ (3)   5. ሁል ጊዜ (4) |  |  |
|  | ውጤት (ከ 8) |  | |

**ምዕራፍ አራት፡ WHOQOL-BREF**

| ተ.ቁ | ጥያቄ | | በጣም መጥፎ | | መጥፎ | | ጥሩም መጥፎም ያልሆነ | | ጥሩ | | | በጣም ጥሩ | |
| --- | --- | --- | --- | --- | --- | --- | --- | --- | --- | --- | --- | --- | --- |
|  | አጠቃላይ የህይወትዎን ሁኔታ እንዴት ይገልጹታል? | | 1 | | 2 | | 3 | | 4 | | | 5 | |
| ተ.ቁ | ጥያቄ | | በጣም ደስተኛ አይደለሁም | | ደስተኛ አይደለሁም | | ደስተኛ ነኝ ወይም አይደለሁም ማለት አልችልም | | ደስተኛ ነኝ | | | በጣም ደስተኛ ነኝ | |
|  | ምን ያህል በጤናዎ ደስተኛ ነዎት? | | 1 | | 2 | | 3 | | 4 | | | 5 | |
| የሚከተሉት ጥያቄዎች ባለፉት ሁለት ሳምንታት ዉስጥ **አንዳንድ ነገሮችን እንዴት እንዳሳለፉ** የሚጠይቁ ናቸዉ፡፡ | | | | | | | | | | | | | |
| ተ.ቁ | ጥያቄ | | ምንም | | በትንሹ | | መካከለኛ/  በመጠኑ | | በጣም | | እጅግ በጣም | | |
|  | ምን ያህል አካላዊ ህመም መስራት ያለብዎን ነገር እንዳያከናዉኑ ከልክሎዎታል? | | 1 | | 2 | | 3 | | 4 | | 5 | | |
|  | ከህመምዎ ጋር የተያያዘ አካለዊ ችግር ምን ያህል ያሳስበዎታል? | | 1 | | 2 | | 3 | | 4 | | 5 | | |
|  | የቀን ከቀን ስራዎን ለማከናወን ምን ያህል የህክምና እርዳታ ያስፈልጎታል? | | 1 | | 2 | | 3 | | 4 | | 5 | | |
|  | ምን ያህል በህይወትዎ ደስተኛ ነዎት? | | 1 | | 2 | | 3 | | 4 | | 5 | | |
|  | ምን ያህል ህይወትዎ ትርጉም ያለዉ እንደሆነ ይሰማዎታል? | | 1 | | 2 | | 3 | | 4 | | 5 | | |
|  | ምን ያህል ሰዎች በህመምዎ ምክንያት የሚወቅስዎት ያሳስብዎታል? | | 1 | | 2 | | 3 | | 4 | | 5 | | |
|  | ምን ያህል ነገን/መጪዉን ጊዜ/ ይፈሩታል? | | 1 | | 2 | | 3 | | 4 | | 5 | | |
|  | ምን ያህል ስለ ሞት ይጨነቃሉ? | | 1 | | 2 | | 3 | | 4 | | 5 | | |
|  | ምን ያህል ለነገሮች ትኩረት ይሠጣሉ? | | 1 | | 2 | | 3 | | 4 | | 5 | | |
|  | በቀን ከቀን ህይወትዎ ምን ያህል ደህንነት ይሠማዎታል? | | 1 | | 2 | | 3 | | 4 | | 5 | | |
|  | አከባቢዎ/ ዙሪያዎ ምን ያህል ጤናማ ነዉ? | | 1 | | 2 | | 3 | | 4 | | 5 | | |
| ያሚከተሉት ጥያቄዎች **ባለፉት ሁለት ሳምንታት** ውስጥ **አንዳንድ ነገሮችን እንዴት እንዳከናወኑ** የሚጠይቁ ናቸዉ፡፡ | | | | | | | | | | | | | |
| ተ.ቁ | ጥያቄ | | ምንም | | በትንሹ | | መካከለኛ/  በመጠኑ | | ብዙ ጊዜ | | ሙሉ በሙሉ | | |
|  | የቀን ከቀን ኑሮዎን ለማከናወን የሚሆን በቂ የሰውነት አቅም አለዎት? | | 1 | | 2 | | 3 | | 4 | | 5 | | |
|  | አሁን ያለዎትን የሠዉነት አካለዊ ሁኔታ አሜን ብለዉ የሚቀበሉት ነዉ? | | 1 | | 2 | | 3 | | 4 | | 5 | | |
|  | ቀንዎችን ለማሣለፍ የሚሆን በቂ ገንዘብ አለዎት? | | 1 | | 2 | | 3 | | 4 | | 5 | | |
|  | ምን ያህል በሚያዉቋቸዉ ሰዎች ዘንድ ተቀባይነት እንዳለዎት ይሠማዎታል? | | 1 | | 2 | | 3 | | 4 | | 5 | | |
|  | ለቀን ከቀን ኑሮዎ የሚያስፈልግዎን መረጃ ምን ያህል ያገኛሉ? | | 1 | | 2 | | 3 | | 4 | | 5 | | |
|  | ምን ያህል የትርፍ ጊዜ ስራዎችን ለመስራት እድሉ አለዎት? | | 1 | | 2 | | 3 | | 4 | | 5 | | |
| ተ.ቁ | ጥያቄ | | በጣም መጥፎ | | መጥፎ | | ጥሩም መጥፎም ያልሆነ | | ጥሩ | | በጣም ጥሩ | | |
|  | በዙሪያዎ ለመንቀሳቀስ ምን ያህል ይችላሉ? | | 1 | | 2 | | 3 | | 4 | | 5 | | |
| የሚከተሉት ጥያቄዎች **ባለፉት ሁለት ሳምንታት** በህይወትዎ ዉስጥ ባሉ በተለያዩ ነገሮች ምን ያህል ደስተኛ እንደሆኑ/ እርካታ እንዳገኙ የሚጠይቁ ናቸዉ፡፡ | | | | | | | | | | | | | |
| ተ.ቁ | ጥያቄ | | በጣም ደስተኛ አይደለሁም | | ደስተኛ አይደለሁም | | ደስተኛ ነኝ ወይም አይደለሁም ማለት አልችልም | | | ደስተኛ ነኝ | | | በጣም ደስተኛ ነኝ |
|  | ምን ያህል በእንቅልፍዎ ደስተኛ ነዎት? | | 1 | | 2 | | 3 | | | 4 | | | 5 |
|  | የቀን ከቀን ስራዎን በማከናወን ረገድ ምን ያህል ደስተኛ ነዎት? | | 1 | | 2 | | 3 | | | 4 | | | 5 |
|  | ስራን ለማከናወን ባለዎት አቅም ምን ያህል ደስተኛ ነዎት? | | 1 | | 2 | | 3 | | | 4 | | | 5 |
|  | በራስዎ ምን ያህል ደስተኛ ነዎት? | | 1 | | 2 | | 3 | | | 4 | | | 5 |
|  | ከሰዎች ጋር ባለዎ የግል ግንኙነት ምን ያህል ደስተኛ ነዎት? | | 1 | | 2 | | 3 | | | 4 | | | 5 |
|  | በግብረ ስጋ ግኑኝነት ህይወትዎ ምን ያህል ደስተኛ ነዎት? | | 1 | | 2 | | 3 | | | 4 | | | 5 |
|  | ከጓደኞችዎ በሚያገኙት እገዛ ምን ያህል ደስተኛ ነዎት? | | 1 | | 2 | | 3 | | | 4 | | | 5 |
|  | በሚኖሩት ቤት ምን ያህል ደስተኛ ነዎት? | | 1 | | 2 | | 3 | | | 4 | | | 5 |
|  | የጤና አገልግሎት በማግኘት ረገድ ምን ያህል ደስተኛ ነዎት? | | 1 | | 2 | | 3 | | | 4 | | | 5 |
|  | በትራንስፖርትዎ ምን ያህል ደስተኛ ነዎት? | | 1 | | 2 | | 3 | | | 4 | | | 5 |
| የሚከተለዉ ጥያቄ **ባለፉት ሁለት ሳምንታት** ውስጥ አንዳንድ ነገሮች ምን ያህል ጊዜ እንደተሰማዎት ወይም እንዳጋጠመዎት የሚጠይቅ ነው፡፡ | | | | | | | | | | | | | |
| ተ.ቁ | ጥያቄ | ምንም ጊዜ | | እንብዛም | | ብዙ ጊዜ | | በጣም ብዙ ጊዜ | | | ሁል ጊዜ | | |
|  | ምን ያህል ጊዜ መጥፎ የሆኑ ስሜቶች ተሰምቶዎታል? (ለምሳሌ ድብርት፣ ጭንቀት፣ተስፋ መቁረጥ የመሳሰሉት) |  | |  | |  | |  | | |  | | |
