# Supplementary material for: Health related quality of life of people receiving highly active antiretroviral therapy in Southwest Ethiopia
Source: PLoS One. 2020 Aug 20;15(8):e0237013. doi: 10.1371/journal.pone.0237013 (PMC7446831; doi:10.1371/journal.pone.0237013)
Supplement: Attachment — Submitted filename: Response to Reviewers .docx [file pone.0237013.s002.docx]

Response to Reviewers’

|  | Reviewers’/editorial comments | Actions taken by authors |
| --- | --- | --- |
| Reviewer 1 | How is the sampling done? is it convenience of random? the duration of mar 4 to apr 1 is of concern as well. it is a little too short combined with convenience sampling, the type of patients surveyed may have a strong responder bias. | Participants who fulfil the inclusion criteria where recruited consecutively. The natural flow patients in the facility is more of random as the chronicity and non-seasonality of the disease, thus this sampling process can represent the target population, less likely to produce bias [1]. The issue of study period is acknowledged under limitation. |
|  | Is the patient reported outcome like MMAPS and WHOQOL-BREF validated in your country? | Yes, these tools are validated and widely used in Ethiopia |
|  | There is much more females than males. is the proportion accurate of your country pls? | In our study, 63.8% patents were females as stated in the result section. This figure represents the picture of the country where females are more affected by HIV than male. According to UNAIDS, 63.08% of PLWHA in Ethiopia are female (UNAIDS, 2018. <https://www.unaids.org/en/regionscountries/countries/ethiopia>) |
| Reviewer 2 | Formatting the manuscript | Corrected according to the journal style |
|  | Minor overlapping | The overlap is on the standard definition of QoL, taken from WHO definition with proper citation. |
|  | Under the Introduction, the authors need to discuss recent global landmark studies on HIV and QoL. | Revised accordingly and as per the comment |
|  | Under the Introduction, the authors stated, "ART may indeed prolong life but may only do so at considerable cost to the QoL of PLWHA (15)". This statement requires further elaboration. | the statement is further elaborated and revised as per the comment |
|  | Under the discussion, the authors stated "The current study found that patients who ever used substances like alcohol, cigarette and khat (AOR=0.39, 95% CI=0.19-0.82) has less likely to have high global quality of life score compared to those patients who never used substances." | We revised according to the comment |
| Editorial | Please describe how verbal consent was documented and witnessed. | As the data collection process is an interviewer administrator questionnaire, consent is requested and documented prior to interview. |
|  | Please include additional information regarding the survey or questionnaire used in the study | We used standard tools (i.e. WHOQOL-HIV-BREF and MMAPS-8) together with self-developed questionnaire to collect data as stated in the method section. The tool is included as supplementary |
|  | Please amend your current ethics statement to confirm that your named institutional review board or ethics committee specifically approved this study | This is included under method section. |
|  | Please provide the participation rate for this study to better allow readers to appreciate the proportion of the sampling frame that agreed to review. This should also be included in the discussion as a limitation. | We included response rate in result section, and a statement of limitation at the last paragraph of discussion |
|  | Please provide a reference for the QOL domain ranges of low/medium/high and the HAART score ranges of high/moderate/low adherence | We have cited references for the cut-off points |
|  | It is unclear if participants' pill counts were used or whether adherence was purely determined based on the MMAPS-8; please clarify. | As stated in the method section we used MMAPS-8 to measure adherence, hence pill counts were not done |
|  | Please spell out corrected and adjusted odds ratios before using the short forms | We edited it accordingly |
|  | Please highlight if all participants provided written informed consent, or if consent was waived | The research involves very low risk harm to participants, verbal consent was taken. |
|  | The manuscript may benefit from another proof-read to correct the typographical and grammatical errors (e.g., the use of articles before nouns, etc.) throughout the manuscript. | We have thoroughly edited the manuscript |

**References**

1	Geldsetzer P, Fink G, Vaikath M, Bärnighausen T. Sampling for Patient Exit Interviews: Assessment of Methods Using Mathematical Derivation and Computer Simulations. Health Serv Res. 2018;53(1):256-72.
