# Supplementary material for: Health related quality of life of people receiving highly active antiretroviral therapy in Southwest Ethiopia
Source: PLoS One. 2020 Aug 20;15(8):e0237013. doi: 10.1371/journal.pone.0237013 (PMC7446831; doi:10.1371/journal.pone.0237013)
Supplement: Attachment — Submitted filename: Response to editorial comments.docx [file pone.0237013.s003.docx]

Response to editorial comments

| Comments | Author action |
| --- | --- |
| The cut-off point for categorising HRQOL | revised using sample mean as a reference |
| Response rate (240 out of 311) | The reasons for decline and exclusion is described. With regard to power, we believe that 240 has statistically sufficient to detect difference. This is evident in our result with narrow confidence interval. But this cannot preclude for all variables, particularly with many categories. This is stated as limitation |
| limitation of MMPAS-8 over pill count | acknowledged as limitation |
| Interviewer administered over self-administered | This option used because of high rate of illiteracy, with the acknowledgement of its potential influence on social desirability bias as limitation |
| Demographic distribution of sample relative to the sampling frame | Our study characteristics is almost in line the sociographic of participants previous conducted in the same sampling frame (<https://www.wjahr.com/admin/assets/manuscripts/0665ca7fc86a8fd3a489351128a578a5.pdf>) and with the national data where more female are affected by HIV (<https://www.unaids.org/en/regionscountries/countries/ethiopia>)). |
